# A Study of the Combined Effects of Physical Activity and Air Pollution on Mortality in Elderly Urban Residents: The Danish Diet, Cancer, and Health Cohort

**DOI:** 10.1289/ehp.1408698

**Published:** 2015-01-27

**Authors:** Zorana Jovanovic Andersen, Audrey de Nazelle, Michelle Ann Mendez, Judith Garcia-Aymerich, Ole Hertel, Anne Tjønneland, Kim Overvad, Ole Raaschou-Nielsen, Mark J. Nieuwenhuijsen

**Affiliations:** 1 Center for Epidemiology and Screening, Department of Public Health, University of Copenhagen, Copenhagen, Denmark; 2 Danish Cancer Research Center, Danish Cancer Society, Copenhagen, Denmark; 3 Centre for Environmental Policy, Imperial College London, London, United Kingdom; 4 Department of Nutrition, University of North Carolina at Chapel Hill, Chapel Hill, North Carolina, USA; 5 Center for Research in Environmental Epidemiology (CREAL), Barcelona, Spain; 6 Universitat Pompeu Fabra, Barcelona, Spain; 7 CIBER Epidemiología y Salud Pública (CIBERESP), Barcelona, Spain; 8 Department of Environmental Science, Aarhus University, Roskilde, Denmark; 9 Section for Epidemiology, Department of Public Health, Aarhus University, Aarhus, Denmark; 10 Department of Cardiology, Aalborg University Hospital, Aalborg, Denmark

## Abstract

**Background:**

Physical activity reduces, whereas exposure to air pollution increases, the risk of premature mortality. Physical activity amplifies respiratory uptake and deposition of air pollutants in the lung, which may augment acute harmful effects of air pollution during exercise.

**Objectives:**

We aimed to examine whether benefits of physical activity on mortality are moderated by long-term exposure to high air pollution levels in an urban setting.

**Methods:**

A total of 52,061 subjects (50–65 years of age) from the Danish Diet, Cancer, and Health cohort, living in Aarhus and Copenhagen, reported data on physical activity in 1993–1997 and were followed until 2010. High exposure to air pollution was defined as the upper 25th percentile of modeled nitrogen dioxide (NO_2_) levels at residential addresses. We associated participation in sports, cycling, gardening, and walking with total and cause-specific mortality by Cox regression, and introduced NO_2_ as an interaction term.

**Results:**

In total, 5,534 subjects died: 2,864 from cancer, 1,285 from cardiovascular disease, 354 from respiratory disease, and 122 from diabetes. Significant inverse associations of participation in sports, cycling, and gardening with total, cardiovascular, and diabetes mortality were not modified by NO_2_. Reductions in respiratory mortality associated with cycling and gardening were more pronounced among participants with moderate/low NO_2_ [hazard ratio (HR) = 0.55; 95% CI: 0.42, 0.72 and 0.55; 95% CI: 0.41, 0.73, respectively] than with high NO_2_ exposure (HR = 0.77; 95% CI: 0.54, 1.11 and HR = 0.81; 95% CI: 0.55, 1.18, *p*-interaction = 0.09 and 0.02, respectively).

**Conclusions:**

In general, exposure to high levels of traffic-related air pollution did not modify associations, indicating beneficial effects of physical activity on mortality. These novel findings require replication in other study populations.

**Citation:**

Andersen ZJ, de Nazelle A, Mendez MA, Garcia-Aymerich J, Hertel O, Tjønneland A, Overvad K, Raaschou-Nielsen O, Nieuwenhuijsen MJ. 2015. A study of the combined effects of physical activity and air pollution on mortality in elderly urban residents: the Danish Diet, Cancer, and Health cohort. Environ Health Perspect 123:557–563; http://dx.doi.org/10.1289/ehp.1408698

## Introduction

Regular physical activity has many health benefits including reduced all-cause mortality along with a reduced risk of cardiovascular disease, cancer, and diabetes (Johnsen et al. 2013; Samitz et al. 2011; Schnohr et al. 2006; Woodcock et al. 2011). Exposure to air pollution may adversely affect human health by triggering or exacerbating respiratory and cardiovascular conditions, certain cancers, and possibly diabetes, leading to premature mortality (Beelen et al. 2014a; Hoek et al. 2013; Raaschou-Nielsen et al. 2012, 2013).

Declining rates of physical activity (Brownson et al. 2005) have given rise to population-level health initiatives including promotion of active transport in cities, encouraging a shift from car use to cycling and walking (de Nazelle et al. 2011). These initiatives are also highly relevant as a solution to other urban challenges such as traffic congestion, air pollution, and greenhouse-gas emission problems in major cities. One of the major challenges to active transport initiatives and other efforts to promote exercise is the trade-off between the health benefits of increased physical activity and potential harms due to amplified exposure to air pollution during outdoor physical activity in urban areas (Rojas-Rueda et al. 2011, 2012, 2013; Woodcock et al. 2014). Increased respiratory uptake and deposition of air pollutants in the lung due to higher minute ventilation during physical exercise may amplify harmful effects of air pollution, even in young and healthy individuals (Giles and Koehle 2014; Strak et al. 2010). In controlled, real-life exposure studies, reduced lung function has been reported in association with walking on a busy street in London (McCreanor et al. 2007; Zhang et al. 2009), running near heavy traffic close to major highway (Rundell et al. 2008), cycling during rush hour on a heavy-traffic route (Strak et al. 2010), or hiking on high air pollution days (Korrick et al. 1998). Similarly, exposure to air pollution and exercise in a controlled setting was reported to alter markers of vascular impairment, arterial stiffness, and vascular reactivity and to reduce exercise performance (Cutrufello et al. 2012; Lundbäck et al. 2009; Rundell and Caviston 2008; Shah et al. 2008) and alter immune function (Chimenti et al. 2009). These studies documented evidence of acute adverse health effects of short-duration exposures to high levels of air pollution during exercise, which seem to be transient and reversible after exercise, at least in young healthy individuals.

One study examined whether exercise modified associations of acute (same-day) exposure to air pollution with mortality in Hong Kong, and reported that regular exercise may reduce premature death attributable to air pollution in elderly subjects (Wong et al. 2007). A cohort study in children reported that participating in sports was associated with development of asthma in children residing in areas with high ozone, but not in areas with low ozone levels (McConnell et al. 2002). These studies implied that there is a potential interaction between physical activity and air pollution, yet no cohort study in adults has explored whether long-term exposure to air pollution modifies beneficial health effects of physical activity on mortality.

In a large prospective urban cohort, we studied whether reductions in mortality linked to regular outdoor leisure-time and transport-related physical activity in terms of doing sports, cycling, gardening, and walking (Johnsen et al. 2013) were modified by long-term exposure to high levels of air pollution at residence.

## Materials and Methods

*Design and study population*. This study was based on the Danish Diet, Cancer, and Health cohort, described in detail elsewhere (Tjønneland et al. 2007). In brief, the cohort consists of 57,053 men (48%) and women (52%) born in Denmark, 50–64 years of age, living in Copenhagen or Aarhus, with no previous cancer diagnosis at the time of enrollment (1993–1997). The participants completed an extensive questionnaire on diet, smoking, alcohol consumption, education, occupation, physical activity, history of diseases and medication, and other health-related items, and provided blood samples, blood pressure, and height and weight measurements at enrollment. Relevant Danish ethical committees and data protection agencies approved the study, and written informed consent was provided by all participants.

*Mortality definition*. Each cohort member was followed up in the Danish Register of Causes of Death (Helweg-Larsen 2011) until 31 December 2009, using a unique personal identification number. On the basis of the underlying cause of death, we defined total mortality as all mortality from natural causes [*International Classification of Diseases, 10th Revision* (ICD-10) codes A00–R99], cancer mortality (C00–C97), cardiovascular mortality (I00–I99), respiratory mortality (J00–J99), and diabetes mortality (E10–E14). We extracted the date of emigration or disappearance and the addresses of all cohort members from the Central Population Registry (Pedersen 2011) using their personal identification numbers.

*Physical activity*. Physical activity was assessed by a self-administered, interviewer-checked questionnaire in which leisure time and transport-related (e.g., to and from work, shopping) physical activity was reported as hours per week spent on sports, cycling, gardening, walking, housework (cleaning, shopping), and “do-it-yourself” activities (e.g., house repair). Data were collected separately for winter and summer of the previous year, and the two values were averaged, so that being active implies at least half an hour spent on a specific activity per week. The physical activity questions have been validated in two studies that found high correlation between self-reported physical activity estimates with the accelerometer measurements of total metabolic equivalent in 182 subjects (Cust et al. 2008) and with combined heart rate and movement sensing measurements in 1,941 subjects (InterAct Consortium et al. 2012). We focused in this study on sports, cycling, and gardening, which were previously associated with lower mortality in the same cohort (Johnsen et al. 2013), and additionally walking at least half an hour per week, which is relevant as an outdoor physical activity pertinent to exposure to air pollution. A previous analysis of data from the cohort indicated that accounting for the amount of physical activity did not substantially alter associations with mortality when activity was dichotomized as any participation versus none (Johnsen et al. 2013). Therefore, our main analyses focused on the estimated effect of participation (yes/no) in sports, cycling, gardening, and walking on mortality, whereas associations with the amount of cycling (categorized as does not cycle, 0.5–4 hr/week, or > 4 hr/week) were estimated in sensitivity analyses.

*Air pollution exposure*. The outdoor concentration of nitrogen dioxide (NO_2_) was calculated at the residential addresses of each cohort member with the Danish AirGIS dispersion modeling system (http://www.dmu.dk/en/air/models/airgis/). AirGIS is based on a geographical information system (GIS) and provides estimates of traffic-related air pollution with high temporal (1-year averages) and spatial (address-level) resolution. AirGIS is a validated model: High correlation was found between AirGIS estimated and measured NO_2_ values (Raaschou-Nielsen et al. 2000), which has been used in a number of studies (Andersen et al. 2012b, 2012c; Raaschou-Nielsen et al. 2011, 2012, 2013) and is described in more detail in the Supplemental Material, “AirGIS Human Exposure Modelling System.” We used the mean of annual concentrations of NO_2_ at residential addresses of each cohort participant since 1971 until the end of follow-up as a proxy of average exposure to traffic-related air pollution during exercise. We defined an indicator variable of high versus moderate/low NO_2_ exposure separated by the 75th percentile of exposure range in the cohort (≥ vs. < 19.0 μg/m^3^).

*Statistical methods*. We used Cox proportional hazards regression with age as the underlying time scale to simultaneously estimate associations between mortality and participation in sports, cycling, gardening, and walking, with separate models used to estimate associations of the four activities with total, cancer, cardiovascular, respiratory, and diabetes mortality, respectively. The follow-up started on the date of enrollment into the cohort (1993–1997) until the date of death, emigration, or 31 December 2009, whichever came first. We fit a crude model adjusted for age (underlying time scale), each of the four domains of physical activity, NO_2_, sex, and year of enrollment into cohort. In addition we fit a fully adjusted model that also included occupational physical activity (sedentary work, standing work, manual work, heavy manual work, or no occupation), smoking status (never, previous, current), lifetime smoking intensity (spline), smoking duration (years), environmental tobacco smoke (indicator of exposure to smoke in the home and/or at work for at least 4 hr/day), alcohol intake (indicator and spline for intensity, grams/day), educational level (< 8, 8–10, or > 10 years of education), fruit and vegetable intake (grams/day), fat intake (grams/day), occupational risk (indicator of a year or longer in an occupation with potential exposure to smoke, particles, fumes or chemicals: mining, rubber industry, tannery, chemical industry, wood-processing industry, metal processing, foundry, steel-rolling mill, shipyard, glass industry, graphics industry, building industry, truck, bus, or taxi driver, manufacture of asbestos or asbestos cement, asbestos insulation, cement article industry, china and pottery industry, painter, welder, hairdresser, auto mechanic), and mean income in the municipality of residence at enrollment (spline), We checked the proportional hazards assumption for all categorical variables by testing for a non-zero slope in a generalized linear regression of the scaled Schoenfeld residuals on functions of time [*estat phtest* command in Stata (StataCorp, College Station, TX, USA)]. We detected violation of proportional hazards assumption by marital status (single, married, divorced, widow/widower) and therefore stratified the model by this variable. Significance level < 0.05 was considered as statistically significant result in all analyses. An additional model was fit in which potentially mediating variables [body mass index (BMI; continuous, kilograms per meter squared), self-reported diagnosis or medication for hypertension and hypercholesterolemia] were added to the full model. Effect modification of associations between the four physical activities (yes/no) and mortality from different causes by exposure to NO_2_ (moderate/high or low) was evaluated by introducing an interaction term into the model, and tested using likelihood ratio tests. Additionally, for cycling, we tested whether there was an interaction between intensity of cycling (> 4 hr/week, 0.5–4 hr/week, does not cycle) and NO_2_ levels, to examine whether there is a dose–response relationship with different levels of NO_2_ [very high, ≥ 23.9 μg/m^3^ (90th percentile of exposure range); moderate, 15.1–23.9 μg/m^3^; low, < 15.1 μg/m^3^ (50th percentile of exposure range)]. We also conducted sensitivity analyses using 1-year mean of NO_2_ at the residential address at enrollment (1993–1997, corresponding to the time period for the self-reported physical activity data) as an alternative proxy of exposure to air pollution. Finally, we conducted two sensitivity analyses on total and respiratory mortality: *a*) with high exposure to air pollution defined as NO_2_ levels above the 90th percentile (23.9 μg/m^3^) of exposure range; and *b*) in a cohort subset consisting of 13,948 subjects living in inner Copenhagen (municipalities of Copenhagen and Frederiksberg)—the most urban part of the cohort, with the highest levels of cycling and air pollution (75th percentile of NO_2_ distribution = 24 μg/m^3^). Results are presented as hazard ratios (HRs) with 95% confidence intervals (CIs), estimated with *stcox* in Stata 11.2.

## Results

Of 57,053 cohort members, 571 were excluded due to cancer diagnosis before baseline, 2 due to uncertain date of cancer diagnosis, 960 for whom an address history was not available for at least 80% of the time between 1971 and recruitment date in the Central Population Registry or their address at baseline could not be geocoded, 948 due to missing air pollution exposure (due to missing traffic counts or other air pollution model input data), and 2,511 due to missing information for a potential confounder or effect modifier, leaving 52,061 cohort members for the study. Excluded subjects did not differ significantly from the rest of the cohort with respect to age, physical activity levels, and education (results not shown).

Study participants were followed for a mean of 13 years, resulting in 677,760 person-years, during which 5,534 (10.6%) died. Of these, 2,864 (50.6%) died from cancer, 1,285 (23.2%) from cardiovascular disease, 354 (6.4%) from respiratory disease, and 122 (2.2%) from diabetes as the underlying cause of death.

Mean age at recruitment was 56.6 years (Table 1). Most of the study subjects participated in physical activity: 54.3% participated in sports, 68.0% cycled, 73.5% gardened, and 93.0% walked. Participation in all physical activities was lower among those who died during follow-up than in the entire cohort (Table 1), with the lowest participation among those who died from respiratory disease and diabetes (Table 2). The mean concentration of NO_2_ at residence was 16.9 ± 5.2 μg/m^3^ for the cohort and 17.9 ± 5.7 μg/m^3^ for the subjects who died during follow-up (Table 1).

**Table 1:** Characteristics of 52,061 participants in the Danish Diet, Cancer, and Health cohort.

Baseline cohort covariates	Total (*n* = 52,061)	Deceased^*a*^(*n* = 5,534)
Age at cohort entry (years)	56.6 ± 4.3	58.5 ± 4.4
Males	24,734 (47.5)	3,292 (59.5)
Participating in sports	28,274 (54.3)	2,189 (39.5)
Cycling	35,385 (68.0)	3,275 (59.2)
Gardening	38,261 (73.5)	3,612 (65.3)
Walking	48,436 (93.0)	5,060 (91.4)
Time/week^*b*^ participating in sports (hr)	2.4 ± 2.3	2.5 ± 2.4
Time/week^*b*^ cycling (hr)	3.2 ± 3.4	3.4 ± 3.6
Time/week^*b*^ gardening (hr)	3.0 ± 3.2	3.7 ± 3.9
Time/week^*b*^ walking (hr)	4.4 ± 4.6	5.1 ± 5.6
Sedentary work	18,711 (35.9)	1,560 (28.2)
Standing work	8,988 (17.3)	857 (15.5)
Manual work	10,506 (20.2)	954 (17.2)
Heavy manual work	2,405 (4.6)	279 (5.0)
Unemployed	11,451 (22.0)	1,884 (34.0)
Never smoked	18,766 (36.0)	1,021 (18.4)
Previously smoked	14,354 (27.6)	1,249 (22.6)
Currently smoke	18,941 (36.4)	3,264 (59.0)
Smoking intensity in ever smokers (g/day)	16.2 ± 10.1	18.8 ± 10.3
Smoking duration in ever smokers (years)	29.6 ± 12.1	35.4 ± 10.9
Exposed to environmental tobacco smoke	33,323 (64.0)	4,304 (77.8)
Consume alcohol	50,897 (97.8)	5,293 (95.6)
Alcohol use in consumers (g/day)	20.9 ± 21.8	27.1 ± 29.9
BMI	26.0 ± 4.0	26.5 ± 4.6
< 8 years of education	17,064 (32.8)	2,349 (42.2)
8–10 years of education	24,066 (46.2)	2,274 (41.1)
≥ 10 years of education	10,931 (21.0)	911 (16.5)
Fruit and vegetable intake (g/day)	350.3 ± 202.7	310.3 ± 203.0
Fat intake (g/day)	76.0 ± 27.0	79.9 ± 29.3
Single	3,067 (5.9)	363 (6.6)
Married	37,454 (71.9)	3,614 (65.3)
Divorced	8,683 (16.7)	1,144 (20.7)
Widow/widower	2,857 (5.5)	413 (7.5)
Risk occupation^*c*^	17,254 (33.1)	2,312 (41.8)
Hypertension	7,671 (15.3)	388 (25.3)
Hypercholesterolemia	3,126 (6.2)	173 (11.3)
NO_2_	16.9 ± 5.2	17.9 ± 5.7
Upper 25th percentile (NO_2_ > 19.0 μg/m^3^)	13,016 (25.0)	1,806 (32.6)
Values are *n* (%) or mean ± SD. ^***a***^Total natural mortality, excluding external cause of death. ^***b***^For those who participate in physical activity. ^***c***^Worked ≥ 1 year in an industry with exposure to smoke, particles, fumes, or chemicals (see “Methods and Materials”).		

**Table 2:** Participation [*n* (%)] in physical activity by cause-specific mortality for 52,061 participants in the Danish Diet, Cancer, and Health cohort.

Physical activity	Total cohort (*n* = 52,061)	Cancer mortality (*n* = 2,864)	Cardiovascular mortality (*n* = 1,285)	Respiratory mortality (*n* = 354)	Diabetes mortality (*n* = 122)
Participating in sports	28,274 (54.3)	1,226 (42.8)	486 (37.8)	99 (28.0)	23 (18.8)
Cycling	35,385 (68.0)	1,801 (62.9)	736 (57.3)	169 (47.7)	57 (46.7)
Gardening	38,261 (73.5)	1,987 (69.4)	843 (65.6)	184 (52.0)	54 (44.3)
Walking	48,436 (93.0)	2,649 (92.5)	1,162 (90.4)	309 (87.3)	107 (87.7)

Statistically significant inverse associations were observed between participation in sports (vs. non participation) and all mortality (Table 3), with fully adjusted HRs of 0.78 (95% CI: 0.73, 0.82) for total mortality, 0.82 (95% CI: 0.76, 0.89), 0.78 (95% CI: 0.69, 0.88), 0.60 (95% CI: 0.47, 0.77), and 0.34 (95% CI: 0.21, 0.55) for cancer, cardiovascular, respiratory, and diabetes mortality, respectively. Statistically significant inverse associations, somewhat weaker than those for participation in sports, were estimated for cycling (vs. not cycling) and gardening (vs. not gardening) with all but cancer mortality: 0.83 (95% CI: 0.78, 0.88) and 0.84 (95% CI: 0.79, 0.89) for total mortality, 0.78 (95% CI: 0.69, 0.88) and 0.82 (95% CI: 0.72, 0.93) for cardiovascular mortality, 0.62 (95% CI: 0.50, 0.77) and 0.63 (95% CI: 0.50, 0.79) for respiratory mortality, and 0.61 (95% CI: 0.42, 0.89) and 0.42 (95% CI: 0.28, 0.62) for diabetes mortality, respectively. Walking (vs. not walking) was statistically significantly inversely associated with respiratory mortality only (HR = 0.71; 95% CI: 0.51, 0.97). All estimates were robust (remained unchanged or were only slightly attenuated) to additional adjustment for BMI, blood pressure, and hypercholesterolemia (data not shown).

**Table 3:** Association [HR (95% CI)] of total and cause-specific mortality with participation (yes/no) in physical activities among 52,061 participants in the Diet, Cancer, and Health cohort.

Physical activity	Main model		Interaction model, fully adjusted^*b*^		*p*‑Value^*c*^
	Crude^*a*^ model	Fully adjusted^*b *^model	Moderate/low NO_2 _(< 19.0 μg/m^3^)	High NO_2 _(≥ 19.0 μg/m^3^)	
Total mortality (*n* = 5,534)
Sports	0.62 (0.59, 0.65)	0.78 (0.73, 0.82)	0.79 (0.74, 0.85)	0.75 (0.67, 0.83)	0.14
Cycling	0.77 (0.73, 0.81)	0.83 (0.78, 0.88)	0.83 (0.77, 0.88)	0.83 (0.75, 0.92)	0.81
Gardening	0.72 (0.68, 0.77)	0.84 (0.79, 0.89)	0.85 (0.78, 0.92)	0.83 (0.75, 0.91)	0.77
Walking	0.91 (0.83, 1.00)	0.97 (0.88, 1.06)	0.96 (0.86, 1.08)	0.95 (0.80, 1.14)	0.79
Cancer mortality (*n* = 2,864)
Sports	0.66 (0.62, 0.72)	0.82 (0.76, 0.89)	0.84 (0.77, 0.92)	0.77 (0.67, 0.89)	0.12
Cycling	0.86 (0.80, 0.93)	0.93 (0.86, 1.01)	0.92 (0.84, 1.01)	0.95 (0.83, 1.10)	0.96
Gardening	0.87 (0.80, 0.94)	0.96 (0.88, 1.04)	1.00 (0.89, 1.11)	0.86 (0.77, 1.02)	0.27
Walking	1.00 (0.87, 1.15)	1.06 (0.93, 1.23)	1.00 (0.88, 1.22)	1.12 (0.85, 1.48)	0.79
Cardiovascular mortality (*n* = 1,285)
Sports	0.61 (0.54, 0.69)	0.78 (0.69, 0.88)	0.76 (0.66, 0.88)	0.80 (0.65, 0.99)	0.88
Cycling	0.73 (0.66, 0.82)	0.78 (0.69, 0.88)	0.83 (0.72, 0.95)	0.70 (0.58, 0.85)	0.16
Gardening	0.85 (0.71, 1.03)	0.82 (0.72, 0.93)	0.85 (0.72, 1.00)	0.77 (0.63, 0.94)	0.55
Walking	0.85 (0.71, 1.03)	0.88 (0.73, 1.07)	0.86 (0.69, 1.00)	0.91 (0.64, 1.28)	0.95
Respiratory mortality (*n* = 354)
Sports	0.40 (0.31, 0.50)	0.60 (0.47, 0.77)	0.65 (0.49, 0.88)	0.50 (0.32, 0.77)	0.70
Cycling	0.54 (0.43, 0.67)	0.62 (0.50, 0.77)	0.55 (0.42, 0.72)	0.77 (0.54, 1.11)	0.09
Gardening	0.50 (0.40, 0.63)	0.63 (0.50, 0.79)	0.55 (0.41, 0.73)	0.81 (0.55, 1.18)	0.02
Walking	0.63 (0.46, 0.86)	0.71 (0.51, 0.97)	0.67 (0.46, 0.97)	0.89 (0.47, 1.67)	0.40
Diabetes mortality (*n* = 122)
Sports	0.28 (0.17, 0.44)	0.34 (0.21, 0.55)	0.41 (0.23, 0.73)	0.24 (0.10, 0.57)	0.22
Cycling	0.58 (0.40, 0.84)	0.61 (0.42, 0.89)	0.58 (0.36, 0.94)	0.66 (0.37, 1.15)	0.93
Gardening	0.33 (0.22, 0.48)	0.42 (0.28, 0.62)	0.44 (0.27, 0.74)	0.37 (0.20, 0.70)	0.79
Walking	0.74 (0.43, 1.28)	0.77 (0.44, 1.33)	0.82 (0.40, 1.67)	0.73 (0.30, 1.73)	0.74
^***a***^Adjusted for NO_2_, sex, calendar year, and mutually for other three physical activities. ^***b***^Adjusted for NO_2_, sex, calendar year, and mutually for other three physical activities, occupational physical activity, smoking status, smoking intensity, smoking duration, alcohol intake, environmental tobacco smoke, education, fruit and vegetable intake, fat intake, risk occupation, mean income in municipality, and stratified by marital status. ^***c***^*p*-Value for interaction.					

There was no statistically significant effect modification of inverse associations between any of the four physical activities and mortality by NO_2_, except for gardening (*p* = 0.02), and borderline significant associations for cycling (*p*-value for interaction 0.09) on respiratory mortality (Table 3). The inverse associations of cycling and gardening with respiratory mortality were stronger among subjects with moderate/low NO_2_ exposure (HR = 0.55; 95% CI: 0.42, 0.72 and HR = 0.55; 95% CI: 0.41, 0.73, respectively) than among those with high NO_2_ exposure (HR = 0.77; 95% CI: 0.54, 1.11 and HR = 0.81; 95% CI: 0.55, 1.18, respectively). Comparable and slightly attenuated effect estimates were observed for all physical activities with a 1-year mean level of NO_2_ at the cohort baseline (data not shown). There was no significant interaction between cycling intensity and NO_2_ when considering the dose–response relationship between increasing levels of cycling intensity and NO_2_ levels, categorized as low, moderate, or high (see Supplemental Material, Table S1). Furthermore, sensitivity analyses showed no effect modification of associations between total and respiratory mortality with any physical activity when exposure to NO_2_ was dichotomized at the 90th percentile (23.9 μg/m^3^) (see Supplemental Material, Table S2), or in the subset of cohort living in inner Copenhagen (see Supplemental Material, Table S3).

## Discussion

Estimates suggesting that leisure-time participation in sports, cycling, and gardening was associated with lower mortality were not significantly modified by exposure to NO_2_ in an urban setting, for total, cancer, cardiovascular, and diabetes mortality. Estimated benefits of cycling and gardening on respiratory mortality were moderately attenuated among those with high levels of NO_2_ exposure compared with moderate or low exposure.

Our finding of significant reductions in total natural and cause-specific mortality related to physical activity confirm existing evidence (Samitz et al. 2011; Woodcock et al. 2011) including Danish data (Johnsen et al. 2013; Schnohr et al. 2006). Estimated benefits of cycling, including cycling to and from work and shopping, were weaker than estimated effects of participating in sports, but were significant, and comparable to the limited evidence. Benefits of cycling estimated in the present analysis were slightly weaker than those earlier reported for cycling to work on all-cause mortality in another cohort in Denmark (relative risk = 0.72; 95% CI: 0.57, 0.91) (Andersen et al. 2000) and comparable to cycling to work in Chinese women on overall mortality [HR = 0.79; 95% CI: 0.61, 1.01 and HR = 0.66; 95% CI: 0.40, 1.07, for 0.1–3.4 and ≥ 3.5 MET (metabolic equivalent)-hr/day, respectively, compared with no cycling] (Matthews et al. 2007). Estimated inverse effects of gardening on mortality were noteworthy, because they are similar in magnitude to those of cycling, whereas weak inverse associations were detected between walking and respiratory mortality only.

Adverse effects of chronic exposure to air pollution on total natural and cardiovascular mortality are well supported (Hoek et al. 2013), and positive associations were also evident in this Danish cohort, where air pollution levels are relatively low (Beelen et al. 2014a, 2014b; Raaschou-Nielsen et al. 2012, 2013). Long-term exposure to air pollution was also associated with diabetes mortality in this cohort (Raaschou-Nielsen et al. 2013), but not with respiratory mortality, in agreement with the recent meta-analyses of 16 European cohorts, including a subset of the cohort in the present analyses (Dimakopoulou et al. 2014). On the other hand, associations of air pollution with incidence of chronic respiratory disease—asthma and COPD (chronic obstructive pulmonary disease)—have been also found in this cohort (Andersen et al. 2011, 2012a).

Our results—that long-term benefits of physical activity on all major types of mortality were not moderated by exposure to high levels of NO_2_—are novel. This may imply that acute stress and damage to the cardiovascular system induced by short-term exposure to air pollution during exercise, in terms of vascular impairment, arterial stiffness, and reduced blood flow, as shown in earlier studies (Lundbäck et al. 2009; Rundell and Caviston 2008; Shah et al. 2008), seem to be transient and reversible and do not abate long-term benefits of physical activity on mortality. Our results may furthermore be explained by the short duration of the physical activities, with mean of 2–3 hr/week for most activities (Table 1); this implies that extra inhaled dose of air pollution during physical activity, which is a function of increased inhalation and duration, is only a small fraction of total inhaled dose of air pollution (Rojas-Rueda et al. 2011), and is therefore not sufficient to increase the risk of premature mortality. Our results are furthermore in line with a study finding significantly lower levels of physical activity on days with poor air quality among respiratory disease patients, but not in cardiovascular patients, who do not seem immediately enough bothered by air pollution to change their outdoor physical activity habits (Balluz et al. 2008; Wells et al. 2012). Our study thus may imply that effects of long-term exposure to NO_2_ and physical activity on overall and cardiovascular mortality are independent of each other, with benefits of outdoor physical activity not being reduced by exposure to NO_2_.

Inverse associations of cycling and gardening with respiratory mortality were closer to the null among subjects with high NO_2_ exposure (0.77; 95% CI: 0.54, 1.11 and 0.81; 95% CI: 0.55, 1.18, respectively) than among those with moderate/low NO_2_ (0.55; 95% CI: 0.42, 0.72 and 0.55; 95% CI: 0.41, 0.73, respectively). Only one similar study exists in a cohort of children, which, consistent with our findings, showed asthma development only in children living in areas with high ozone concentrations, and not in those living in areas with low ozone (McConnell et al. 2002). It is plausible that amplification of lung damage due to greater inhaled doses of air pollution through physical activity in urban areas with high air pollution may moderate the benefits of physical activity, which improves some of the same physiological mechanisms. Earlier studies have shown that hikers with a history of asthma had significantly greater air pollution–related acute reductions in pulmonary functions than did asthma-free hikers (Korrick et al. 1998), and that subjects with moderate asthma had greater acute lung function reductions after walking on a busy street in London than did those with mild asthma (McCreanor et al. 2007; Zhang et al. 2009). However, reduced physical activity was observed on days with poor air quality among respiratory disease patients (Balluz et al. 2008; Wells et al. 2012), but not among cardiovascular disease patients, as noted earlier. This implies an alternative explanation to our findings that reduced benefit from physical activity in subjects residing in areas with high air pollution may be attributable to abstaining from physical activity on days with high air pollution, and not from enhanced negative effects of greater exposure to air pollution during physical activity. Our findings were weakened by the fact that there was no dose–response relationship in reductions of respiratory mortality related to cycling by number of hours spent cycling and by increasing levels of air pollution (see Supplemental Material, Table S1). Although numbers are small in the interaction analyses, the lack of effect of duration of cycling may imply that the cyclists themselves differ from the noncyclists, and that in general, the effects of air pollution are minimal in healthy people. Finally, significant interactions of cycling and gardening with air pollution observed for respiratory mortality (Table 3) could not be reproduced when considering levels of NO_2_ > 24.9 μg/m^3^ (the 90th percentile) as high exposure, or when considering the subset of subjects living in inner Copenhagen, where levels of air pollution and number of people cycling are at the highest in Denmark (see Supplemental Material, Tables S2 and S3). However, these analyses need to be interpreted with caution because in both sensitivity analyses, exposure levels were also increased in the “low” exposure category, possibly obscuring differences in associations between the lower and higher levels of exposure.

In summary, our findings suggest that outdoor physical activity in areas with high air pollution may moderate, but not reverse, the benefits of physical activity on respiratory mortality: Adverse effects of the additional pollutants inhaled over time do not outweigh the benefits of physical activity. Our results, however, need to be reproduced, because of both the small number of people dying from respiratory causes and the sensitivity of these results to the definition of NO_2_ and subcohort. Furthermore, because of the relatively small number of people dying from respiratory causes (6% in this cohort), and assuming that our results are true, reductions in health benefits related to physical activity in areas with high air pollution are rather marginal.

Strengths of our study include a large prospective cohort, with well-defined and validated information on physical activity and air pollution exposure, both of which have been linked to mortality (Johnsen et al. 2013; Raaschou-Nielsen et al. 2012, 2013). We furthermore benefited from the state-of-the art information on individual exposure to NO_2_ with high spatial (address-specific) and temporal (annual mean) resolution, assessed over 35 years. Another strength of this cohort is the very high prevalence of cycling (68%), both leisure and utilitarian (e.g., to work, shopping); this provided the data for evaluation of an interaction of air pollution with this type of physical activity, in contrast to existing studies on cycling to work (Andersen et al. 2000; Matthews et al. 2007; Rojas-Rueda et al. 2011, 2012). Furthermore, this is the first cohort study to evaluate individual-level benefits of physical activity in an urban cohort while also considering individual exposure to air pollution. A study of short-term effects of air pollution on mortality in Hong Kong, which has several-fold higher levels of air pollution than in Copenhagen, found that those who exercised regularly had reduced susceptibility to acute effects of air pollution and lower mortality than those who did not exercise (Wong et al. 2007). Our study provides a novel approach in contrast to existing health impact assessment studies. Our study estimated benefits versus risks of increased physical activity, typically by evaluating active travel policies targeted to shift commuters from car use to cycling, on a population level, based on derivation of risk estimates from different studies and hypothetical scenarios (Andersen et al. 2000; de Hartog et al. 2010; Rojas-Rueda et al. 2011, 2012, 2013).

A weakness of our study is the use of NO_2_ levels at residence as a proxy of average air pollution levels encountered during physical activity. This assumption works well for gardening, which typically occurs at the residence (the exact location of air pollution modeling) but less well for cycling and walking. Given that this cohort consists of older subjects 50–65 years of age at baseline, many of whom retired before study recruitment or during study follow-up, it is reasonable to assume that most of their time walking and cycling had taken place in close proximity to their residence, which may be well represented by air pollution levels at residence. The higher exposure misclassification is expected for cycling than for gardening. Cycling levels were the same for subjects residing in areas with low/moderate and high air pollution levels, 68%. Gardening was more common in subjects living in areas with low/moderate air pollution (79%) than in those living in areas with high air pollution (58%), because rates of house ownership are higher in the suburbs than in the inner city, where pollution is highest. Furthermore, we did not have information on cycling, walking, and exercising habits of cohort participants, and possible behavioral adjustment by those living in areas of high air pollution levels to avoid the most polluted areas, which may bias results. Similarly, participating in sports is a poor proxy of outdoor activity because we do not have information on the type of sports activity or whether it took place outdoors (running), or indoors (e.g., gym, badminton, swimming). Thus, lack of findings of interaction with air pollution and participation in sports may be attributable to exposure misclassification. Many cohort members retired during the study follow-up, implying the possibility of misclassification of exposure over time, if retirement led to considerable changes in cycling after enrollment. Most biking trips (over 65%) in Denmark are undertaken for leisure activities, shopping, and doing errands, with a minority for transport to and back from work (Danish Technical University 2013). Cycling decreases with age in Denmark, but only marginally, by about 10–15% from 50–59 to 60–69 years, due to retirement/decrease in share of cycling trips to work (Vithen 2013).

Another weakness is a lack of data on particulate matter (PM), which was available for only half of the cohort participants living in Copenhagen (Beelen et al. 2014a) and only for 2010, and was therefore not used here. However, NO_2_ and nitrogen oxides were found to correlate strongly with PM in Denmark, with Spearman correlation coefficient of 0.70 for PM_10_ (PM with diameter ≤ 10 μm) and 0.93 for ultrafine fraction of PM (Hertel et al. 2001; Ketzel et al. 2003), implying that similar results for PM would be expected. Furthermore, we have shown earlier that PM_10_ originates largely from long-range transport in Denmark, resulting in smaller spatial variation than ultrafine PM and NO_2_ (Andersen et al. 2007), which originate mainly from traffic. Traffic is the main focus of this study, reflecting air pollution exposures during exercise or commute. Still, NO_2_ serves as a proxy for all traffic-related pollutants, including elemental carbon, nitric oxide, carbon monoxide, ultrafine PM, noise, and possibly road dust.

Another weakness is that DCH cohort participants are likely healthier than the general Danish population, because they are better educated and had higher income than nonparticipants (Tjønneland et al. 2007). Finally, air pollution levels are relatively low in Copenhagen and Aarhus, and these findings need to be reproduced in sites with higher air pollution levels.

Our results are in agreement with a growing number of health impact assessment studies that evaluate the net effects of an increase in cycling at the population level, typically as a shift from car use, and conclude that health benefits due to increased physical activity levels generally outweigh the risks related to increase inhaled air pollution doses during cycling (de Hartog et al. 2010; Rojas-Rueda et al. 2011, 2012; Woodcock et al. 2014).

## Conclusions

Physical activity plays a key role in improving the physiologic mechanisms and health outcomes that exposure to air pollution may exacerbate. This presents a challenge in understanding and balancing the beneficial effects of physical activity in the urban environment with the detrimental effects of air pollution on human health. Our findings suggest that beneficial effects of physical activity on mortality in an urban area with relatively low levels of air pollution are not moderated in subjects residing in areas with the highest levels of air pollution. Estimated benefits of cycling and gardening on respiratory mortality were marginally reduced, but not annulled, for those living in areas with high NO_2_ levels, but these novel results need confirmation. Overall, the long-term benefits of physical activity in terms of reduced mortality outweigh the risk associated with enhanced exposure to air pollution during physical activity.

## Supplemental Material

(334 KB) PDF
