# Supplementary material for: A Study of the Combined Effects of Physical Activity and Air Pollution on Mortality in Elderly Urban Residents: The Danish Diet, Cancer, and Health Cohort
Source: Environ Health Perspect. 2015 Jan 27;123(6):557–63. doi: 10.1289/ehp.1408698 (PMC4455593; doi:10.1289/ehp.1408698)
Supplement: (334 KB) PDF [file ehp.1408698.s001.508.pdf]

Supplemental Material

A Study of the Combined Effects of Physical Activity and Air Pollution on Mortality in Elderly Urban Residents: The Danish Diet, Cancer, and Health Cohort

Zorana Jovanovic Andersen, Audrey de Nazelle, Michelle Ann Mendez, Judith Garcia-Aymerich, Ole Hertel, Anne Tjønneland, Kim Overvad, Ole Raaschou-Nielsen, and Mark J. Nieuwenhuijsen

AirGIS Human Exposure Modelling System

AirGIS (Jensen et al. 2001) is based on a geographical information system (GIS) and used for estimating traffic-related air pollution and has high temporal (an hour) and spatial (individual address) resolution. AirGIS (see: http://AirGIS.dmu.dk) calculates air pollution at a location as the sum of three contributors: (1) local air pollution from street traffic, calculated with the Operational Street Pollution Model (Berkowicz R 2000) (OSPM) from data on traffic (intensity and type), emission factors for each vehicle type and EURO class, street and building geometry, and meteorology; (2) urban background, calculated from a simplified urban background (SUB) procedure (Berkowicz et al. 2008) that takes into account urban vehicle emission density, city dimensions (transport distance), and average building height (initial dispersion height); and (3) regional background, estimated from trends at rural monitoring stations and from national vehicle emissions (Jensen SS 1998). Input data for the AirGIS system come from various sources: a GIS-based national street and traffic database, including construction year and traffic data for the period 1960–2005, (Jensen SS et al. 2009a) and a database on emission factors for the Danish car fleet (Berkowicz R et al. 2006; Ketzel M et al. 2007), with data on light- and heavy-duty vehicles dating back to 1960, built and entered into the emission module of the OSPM. A national GIS database with building footprints supplemented with construction year and building height from the national building and dwelling register, national survey and cadastre data-bases, and a national terrain-evaluation model, provided the correct street geometry for a given year at a given address. With a geocoded address and a year, the starting point is specified in place and time, and the AirGIS system automatically generates street configuration data for the OSPM, including street orientation, street width, building heights in wind sectors, traffic intensity and type, and the other data required for the model. Air pollution is calculated in

2m height at the façade of the building. The AirGIS system has been successfully validated (Berkowicz R 2008; Raaschou-Nielsen O et al. 2000) and used in a number of studies, few early examples listed here (Jensen SS et al. 2009b; Hertel O et al. 2001; Hertel O. et el. 2008).

References

Berkowicz R. 2000. OSPM - A parameterised street pollution model. Environ Monit Assess 65:323-31.

Berkowitz R, Winther M, Ketzel M. 2006. Traffic pollution modeling and emission data. Environ Model Software 21:454-60.

Berkowicz R, Ketzel M, Jensen SS, Hvidberg M, Raaschou-Nielsen O. 2008. Evaluation and application of OSMP for traffic pollution assessment for large number of street locations. Environ Model Software 23:296-303.

Hertel O, Jensen SS, Andersen HV Palmgren F, Wåhlin P, Skov H.. 2001. Human exposure to traffic pollution. Experience from Danish studies. Pure Appl Chem 73:137-145.

Hertel O, Hvidberg M, Ketzel M, Storm L, Stausgaard L.. 2008. A proper choice of route significantly reduces air pollution exposure–a study on bicycle and bus trips in urban streets. Sci Tot Environ 389:58-78.

Jensen SS. Background concentrations for use in the Operational Street Pollution Model (OSPM). 1998. Roskilde, National Environmental Research Institute, Aarhus University. NERI Technical Reports. 234:1-107.

Jensen SS, Berkowicz R, Hansen SH, Hertel O. 2001. A Danish decision-support GIS tool for management of urban air quality and human exposures. Transport Res Part D: Transport Environ 6:229-41.

Jensen SS, Hvidberg M, Pedersen J,Storm L, Stausgaard L, Becker T. et al. . GIS-based national street and traffic data base 1960-2005 (In Danish; English summary). 2009a. Roskilde, National Environmantal Research Institute, Aarhus University. NERI Technical Reports. 678:1-73.

Jensen SS, Larson T, Deepti KC, Kaufman JD. 2009b. Modeling traffic air pollution in street canyons in New York City for intra-urban exposure assessment in the US multi-ethnic study of atherosclerosis and air pollution. Atmos Environ 43:4544-56.

Ketzel M, Omstedt G, Johansson C, During I, Pohjola M, Oettl D, et al. 2007. Estimation and validation of $PM_{2.5}$/PM_{10} exhaust and non-exhaust emission factors for practical street pollution modeling. Atmos Environ 41:9370-85.

Raaschou-Nielsen O, Hertel O, Vignati E, Berkowicz R, Jensen SS, Larsen VB, et al. 2000. An air pollution model for use in epidemiological studies: evaluation with measured levels of nitrogen dioxide and benzene. J Expo Anal Environ Epidemiol 10:4-14.

Table S1. Adjusted associations[a] of total and cause-specific mortality with cycling among 52,061 participants in Diet, Cancer and Health cohort, by intensity of cycling and different levels of NO_2.

**Physical Activity**	**Low NO_2 (< 15.1 µg/m^3) HR (95% CI**	**Moderate NO_2 (15.1-23.9 µg/m^3) HR (95% CI**	**Very high NO_2 (≥ 23.9 µg/m^3) HR (95% CI**	***p-value*[b]**
**Total mortality (*n* = 5,534)**				
Does not cycle	1.00	1.26 (1.15, 1.39)	1.39 (1.22, 1.58)	
Cycles 0.5-4 h/week	0.87 (0.79, 0.95)	1.00 (0.91, 1.10)	1.10 (0.96, 1.26)	
Cycles >4 h/week	0.82 (0.72, 0.93)	1.02 (0.92, 1.14)	1.19 (1.01, 1.40)	*0.52*
**Cancer mortality (*n* = 2,864)**				
Does not cycle	1.00	1.22 (1.07, 1.39)	1.36 (1.13, 1.64)	
Cycles 0.5-4 h/week	0.97 (0.86, 1.10)	1.09 (0.96, 1.23)	1.19 (0.98, 1.45)	
Cycles >4 h/week	0.91 (0.76, 1.08)	1.14 (0.99, 1.33)	1.16 (0.92, 1.47)	*0.71*
**Cardiovascular mortality (*n* = 1,285)**				
Does not cycle	1.00	1.36 (1.13, 1.64)	1.78 (1.39, 2.29)	
Cycles 0.5-4 h/week	0.83 (0.68, 1.01)	1.09 (0.90, 1.31)	1.21 (0.91, 1.61)	
Cycles >4 h/week	0.73 (0.55, 0.96)	0.98 (0.78, 1.23)	1.38 (1.00, 1.91)	*0.78*
**Respiratory mortality (*n* = 354)**				
Does not cycle	1.00	1.02 (0.74, 1.40)	0.73 (0.45, 1.18)	
Cycles 0.5-2 h/week	0.56 (0.39, 0.81)	0.72 (0.51, 1.02)	0.48 (0.26, 0.89)	
Cycles >4 h/week	0.49 (0.28, 0.85)	0.57 (0.37, 0.88)	0.57 (0.29, 1.12)	*0.78*
**Diabetes mortality (*n* = 122)**				
Does not cycle	1.00	1.36 (0.79, 2.37)	1.20 (0.56, 2.53)	
Cycles 0.5-2 h/week	0.69 (0.35, 1.34)	0.86 (0.46, 1.61)	0.69 (0.25, 1.84)	
Cycles >4 h/week	0.55 (0.21, 1.47)	0.75 (0.36, 1.56)	0.56 (0.16, 1.91)	*0.98*

HR hazard ratio; CI confidence interval.

[a]Adjusted for NO_2, gender, calendar year, and mutually for other three physical activities, occupational physical activity, smoking status, smoking intensity, smoking duration, alcohol intake, environmental tobacco smoke, education, fruit and vegetable intake, fat intake, risk occupation, mean income in municipality, and stratified by marital status. [b]*p*-value for interaction.

Table S2. Adjusted Associations[a] of total and respiratory with different types of physical activities in 52,061 participants in Diet, Cancer and Health cohort with definition of high NO_2 above 90th percentile (23.9 μg/m^3).

**Physical Activity**	**Main Model Fully Adjusted HR (95% CI)**	**Interaction Model Moderate/Low NO_2 (< 23.9 μg/m^3) HR (95% CI)**	**Interaction Model High NO_2 (≥ 23.9 μg/m^3) HR (95% CI)**	***p-value*[b]**
**Total natural mortality (*n* = 5,534)**				
Sports	0.78 (0.73, 0.82)	0.78 (0.74, 0.83)	0.74 (0.63, 0.87)	*0.38*
Cycling	0.83 (0.78, 0.88)	0.84 (0.79, 0. 88)	0.81 (0.71, 0.93)	*0.48*
Gardening	0.84 (0.79, 0.89)	0.86 (0.80, 0.92)	0.78 (0.72, 0.91)	*0.79*
Walking	0.96 (0.88, 1.06)	0.96 (0.87, 1.07)	0.95 (0.72, 1.27)	*0.85*
**Respiratory mortality (*n* = 354)**				
Sports	0.60 (0.47, 0.77)	0.62 (0.47, 0.80)	0.57 (0.28, 1.18)	*0.98*
Cycling	0.62 (0.50, 0.77)	0.61 (0.48, 0.77)	0.74 (0.39, 1.38)	*0.62*
Gardening	0.63 (0.50, 0.79)	0.61 (0.48, 0.78)	0.79 (0.42, 1.51)	*0.35*
Walking	0.71 (0.51, 0.97)	0.73 (0.52, 1.02)	0.67 (0.24, 1.83)	*0.65*

HR hazard ratio; CI confidence interval.

[a]Adjusted for NO_2, gender, calendar year, and mutually for other three physical activities, occupational physical activity, smoking status, smoking intensity, smoking duration, alcohol intake, environmental tobacco smoke, education, fruit and vegetable intake, fat intake, risk occupation, mean income in municipality, and stratified by marital status. p[b]*p*-value for interaction.

Table S3. Adjusted associations[a] of total and respiratory mortality with different types of physical activities in 13,948 participants in Diet, Cancer and Health cohort who lived in inner Copenhagen (Frederiksberg and Copenhagen municipalities, with 75th percentile of NO_2 of 24.0 μg /m^3).

**Physical Activity**	**Main Model Fully Adjusted[b] HR (95% CI)**	**Interaction Model Moderate/Low NO_2 (< 24.0 μg/m^3) HR (95% CI)**	**Interaction Model High NO_2 (≥ 24.0 μg/m^3) HR (95% CI)**	***p-value*[b]**
**Total natural mortality (*n* = 2,053)**				
Sports	0.77 (0.70, 0.85)	0.79 (0.70, 0.88)	0.75 (0.62, 0.90)	*0.67*
Cycling	0.86 (0.78, 0.94)	0.88 (0.79, 0.99)	0.82 (0.69, 0.97)	*0.40*
Gardening	0.84 (0.76, 0.92)	0.86 (0.77, 0.95)	0.79 (0.66, 0.94)	*0.51*
Walking	0.90 (0.77, 1.06)	0.89 (0.74, 1.08)	0.90 (0.65, 1.27)	*0.81*
**Respiratory mortality (*n* = 163)**				
Sports	0.52 (0.36, 0.77)	0.58 (0.38, 0.88)	0.41 (0.16, 1.05)	*0.57*
Cycling	0.74 (0.54, 1.02)	0.73 (0.51, 1.04)	0.84 (0.40, 1.76)	*0.72*
Gardening	0.66 (0.47, 0.93)	0.68 (0.46, 0.99)	0.71 (0.32, 1.55)	*0.95*
Walking	0.77 (0.45, 1.29)	0.88 (0.48, 1.61)	0.49 (0.16, 1.52)	*0.36*

HR hazard ratio; CI confidence interval.

[a]Adjusted for NO_2, gender, calendar year, and mutually for other three physical activities, occupational physical activity, smoking status, smoking intensity, smoking duration, alcohol intake, environmental tobacco smoke, education, fruit and vegetable intake, fat intake, risk occupation, mean income in municipality, and stratified by marital status. [b]*p*-value for interaction.
